# Responsiveness and minimal important change for the ProFitMap-neck questionnaire and the Neck Disability Index in women with neck–shoulder pain

**DOI:** 10.1007/s11136-016-1373-8

**Published:** 2016-08-09

**Authors:** Martin Björklund, Birgitta Wiitavaara, Marina Heiden

**Affiliations:** 1 Centre for Musculoskeletal Research, Department of Occupational and Public Health Sciences, University of Gävle, SE-801 76 Gävle, Sweden; 2 Department of Community Medicine and Rehabilitation, Physiotherapy, Umeå University, SE-901 87 Umeå, Sweden

**Keywords:** Validity, Anchor-based, Physical function, Discrimination, Sensitivity, Specificity

## Abstract

**Purpose:**

The aim was to determine the responsiveness and minimal important change (MIC) of the questionnaire ProFitMap-neck that measures symptoms and functional limitations in women with neck pain. The same measurement properties were determined for Neck Disability Index (NDI) for comparison purposes.

**Methods:**

Longitudinal data were derived from two randomized controlled trials, including 103 and 120 women with non-specific neck pain, with questionnaire measurements performed before and after interventions. Sensitivity and specificity to discriminate between improved and not or little changed participants, based on categorization of a global rating of change scale (GRCS), were determined for the ProFitMap-neck indices and NDI by using area under receiver operating characteristic curves (AUC). Correlations between the GRCS anchor and change scores of the questionnaires were also used to assess responsiveness. The change score that showed the highest combination of sensitivity and specificity was set for MIC.

**Results:**

The ProFitMap-neck indices showed similar responsiveness as NDI with AUC exceeding 0.70 (Range: ProFitMap-neck, 0.74–0.83; NDI, 0.75–0.86). The MIC in the two samples ranged between 6.6 and 13.6 % for ProFitMap-neck indices and 5.2 and 6.3 % for NDI. Both questionnaires had significant correlations with GRCS (Spearman’s rho 0.47–0.72).

**Conclusions:**

Validity of change scores was endorsed for the ProFitMap-neck indices and NDI with adequate ability to discriminate between improved and not or little changed participants. Values of minimal important change were presented.

## Introduction

Neck pain is highly prevalent with a reported 1-year prevalence estimated to be 30 to 50 % in the general population [1]. Neck pain also contributes to activity limitations in 11 to 14 % of workers [2]. In the largest group of neck pain patients, the underlying cause of the pain is uncertain [3, 4]; hence, the designation is non-specific neck pain. The alleviation of symptoms and restoration of functional limitations are particularly important for neck pain sufferers without a clear pathophysiology. To evaluate and establish effective treatment and rehabilitation strategies, access to reliable and valid patient-reported outcome measures, i.e., standardized questionnaires measuring specific constructs of interest, is a necessity. There are a number of questionnaires available to measure pain and disability in people with neck pain. However, weaknesses in measurement properties of several questionnaires were recently recognised, and important methodological aspects to improve were, for example, content validity regarding the relevance and comprehensiveness of items and the use of better statistical methods in responsiveness studies [5, 6]. Also, Wiitavaara and co-workers [7] found a low correspondence between neck–shoulder pain questionnaires and the symptoms experienced by the sufferers, implying a questionable content validity of the questionnaires. One potential explanation for this may be that the neck pain sufferers’ experiences are seldom taken into account in the developmental process of the neck–shoulder pain questionnaires [7], even though it is recommended in the literature [6, 8–11].

The Profile Fitness Mapping neck questionnaire (ProFitMap-neck) is a questionnaire developed in collaboration with neck pain patients, designed to assess symptoms and functional limitations in people with neck pain [12]. It consists of a functional limitation scale and a symptom scale of which the latter is subdivided in separate indices for the intensity and frequency of symptoms. The two scales can also be combined in a compound total score. The content of ProFitMap-neck symptom scale had the best correspondence with experienced symptoms among subjects with chronic neck pain, compared with 9 other neck-specific questionnaires [7]. The function scale of ProFitMap-neck has not been compared in the same way, but items of this scale have shown associations with sensorimotor function tests in different groups of people with neck pain [13–16]. The overall validity and reliability of the questionnaire has been tested on patients with chronic whiplash-associated disorders, as well as chronic non-traumatic non-specific neck pain [12]. However, the validation study of Björklund and co-workers [12] had a cross-sectional design that assessed validity of single scores. To evaluate the ability of an instrument to detect change over time in the construct to be measured, a measurement property referred to as responsiveness [17], longitudinal study designs are necessary.

An issue related to responsiveness concerns the interpretation of a change score, i.e., the change of a score from baseline to a follow-up. It is important to know if a change score of an instrument reflects a change in the patient’s status that he/she would consider important. The cut-off score with the best discriminative ability between patients that have improved and not improved is often referred to as the minimal important change (MIC) of the instrument, defined as the smallest measured change score that patients perceive to be important [17, 18]. The knowledge of a questionnaire’s responsiveness and MIC is crucial for its use in the evaluation of treatment and rehabilitation. In clinical practice, it can be used to judge whether a patient has reached a change of importance, and in research, the measurement properties are useful for the analysis and interpretation of study results. The primary aim of the present study was to determine the responsiveness and MIC of the ProFitMap-neck and the Neck Disability Index (NDI) [19] in women with chronic non-specific neck–shoulder pain. A secondary aim was to compare the responsiveness between ProFitMap-neck and NDI. We chose to compare with NDI since it is the most frequently used and evaluated neck-specific questionnaire [5, 20, 21].

## Materials and methods

Data for the current study were derived from two randomized controlled trials (ISRCTN trial registration numbers ISRCTN92199001 [22]) and ISRCTN49348025 [23]. Both trials had an observer-blinded three-arm parallel group design with baseline measures and follow-ups 1 week, 6 months and 12 months after an 11-week intervention. For the purpose of the current study, only the measurements at baseline and 1 week after intervention were used. Both trials were approved by the Ethical Review Board in Uppsala, Sweden, and informed consent was obtained from all individual participants included in the study. The two trials with their adherent samples will from here on be called trial I—sample I [22] and trial II—sample II [23], respectively.

## Trial I

The purpose of trial I was to evaluate the effects of neck coordination exercise, compared to either strength training for the neck and shoulder regions or massage treatment, in 108 women with non-specific neck–shoulder pain [22]. The inclusion criteria for the study were women, age 25–65 years, with more than 3 months of non-specific neck pain with the neck region indicated as the dominant pain area on a pain drawing [24] and disability with limitations in performing everyday activities involving the neck, shoulders and arms according to DASH [25]. Excluded were those that had trauma-related neck pain, diagnosis of a psychiatric, rheumatic, neurological, inflammatory, endocrine or connective tissue disease, fibromyalgia, cancer, stroke, cardiac infarction or diabetes type I, surgery or fracture to the back, neck, or shoulder in the last 3 years, shoulder luxation in the last year or reported strenuous exercise >3 times/week during the last 6 months. All interventions comprised of 22 individually supervised treatment sessions. The neck coordination exercise was performed with a training device that participants wore on their head [26]. The exercise task was to control, through visual feedback via mirrors, the movement of a metal ball placed on the device with the aim to improve the fine movement control of the cervical spine. The strength training intervention consisted of isometric and dynamic exercises for the neck- and shoulder muscles, inspired by the training programme of Ylinen and co-workers [27]. The massage treatment consisted of classical massage for the back, neck and shoulders.

## Trial II

In trial II, the purpose was to evaluate individualized treatment compared to non-individualized treatment or treatment as usual (participants received no treatment from the study and no restriction to what they were allowed to do) in 120 women with non-specific neck–shoulder pain [23]. The inclusion and exclusion criteria were the same as in trial I with the following exceptions: The age span in trial II was 20–65 years, pain duration was minimum 6 weeks, and participants were required to have between mild and severe disability according to NDI [19] (participants did not answer DASH in trial II) and impaired capacity to work due to neck problems [28]. Also, in trial II, strenuous exercise was not an exclusion criteria, but concurrent low back pain was. Participants of the two intervention groups received treatments two to three times per week for a period of 11 weeks. The individualized treatment was tailored to the individuals’ functional limitations and symptoms, as decided from a decision model comprising the five categories cervical mobility, neck–shoulder strength and motor control, eye–head–neck control, trapezius myalgia and cervicogenic headache. The non-individualized treatment included the same available treatment components but applied quasi-randomly [23].

## Measurements

In both trial I and II, the participants answered a comprehensive set of questionnaires at each test occasion. This set included ProFitMap-neck [12] NDI [29] and a global rating of change scale (GRCS, only administered after intervention). In the present study, the GRCS is used as a comparator instrument and external anchor of change in relation to ProFitMap-neck and NDI.

### Profile Fitness Mapping neck questionnaire

The two original scales of ProFitMap-neck, the functional limitation scale (function index) and the symptom scale (intensity index and frequency index), consist of 20 and 27 items. After a recent validation study [12], revisions of the scales were suggested by reducing items of the scales to 18 and 26, respectively. In the present study, the revised scales are used. Each item has six response alternatives with the following ranges: Function index (*how do you manage to*) from “very good, no problem, very satisfying, very likely” to “very bad, very difficult/impossible, very dissatisfying, very unlikely”; Symptom scale, intensity index (*how much*) from “nothing/none at all” to “almost unbearable/unbearable, all/maximally”; Symptom scale, frequency index (*how often*) from “never/very seldom” to “very often/always”. The index scores are normalized 0–100 with higher scores reflecting better function/better health (function index) and less symptoms/better health (symptom indices intensity index and frequency index). In addition, a total score is calculated as the average of the three indices. For a detailed description of items and method of index score calculation, see appendix in [12]. The ProFitMap-neck indices have shown good internal consistency in three different neck pain samples, with Cronbach’s α ranging between 0.88 and 0.96, and ICC test–retest reliability ranging between 0.80 and 0.91 [12].

### Neck Disability Index

The NDI measures symptoms and disability related to neck pain [19]. It contains 10 items about pain intensity, concentration, headache and activities of daily living. The items have six response alternatives ranging from no disability (0) to total disability (5), thus the sum score ranges from 0 to 50. In the present study, the NDI index was normalized 0–100 with higher scores reflecting higher levels of disability. A recent review of psychometric properties of neck-specific questionnaires [5] concluded that the NDI is the most frequently validated neck questionnaire and that it has limited positive content validity, correlates with questionnaires measuring pain/physical functioning (r = 0.53–0.70), and moderate evidence for responsiveness. However, the reliability of NDI may not be sufficient [30], and the estimation of MIC seems uncertain with widely differing estimates between studies (for references, see [5]). Hence, the use of NDI in the current study might also contribute with more knowledge about the MIC of NDI.

### Global rating of change scale

The global rating of change scale (GRCS) used in trial I and II was a single question, asking for the participant’s change after treatment, with responses on a balanced 7-point Likert scale: 1. Very much worse; 2. Much worse; 3. Minimally worse; 4. No change; 5. Minimally improved; 6. Much improved; 7. Very much improved. The Initiative on Methods, Measurement, and Pain Assessment in Clinical Trials (IMMPACT) recommends this 7-point scale (referring to it as the Patient Global Impression of Change Scale) to be a core outcome measure of global improvement in chronic pain clinical trials [31]. There are examples in the literature of GRCS with various numbers of response alternatives, usually ranging from 3 to 15 [32], but GRCS with 7 to 11 points seems to be most appropriate when taking reliability, discriminative ability and patient preferences into account [33].

The wording of the GRCS at evaluation one week after intervention was “Compared to before the treatment of the study started, my overall status is now” (trial I), and “Compared to before the treatment of the study started, my status regarding my neck–shoulder problems is now” (trial II). For the purpose of the present study, the GRCS was used as the external criterion of *improved* (participants rating 6 and 7) and *no or little change* (participants rating 3, 4 and 5) for the determination of responsiveness and MIC [34, 35]. Participants with GRCS rating 1 and 2 were excluded from the analysis [35].

### Statistical analysis

As described previously, all questionnaire indices were expressed as a percentage of the maximum possible score, where a higher percentage reflects better health/function/less symptoms in ProFitMap-neck indices and more disability in NDI. If an item was omitted by a respondent, the maximum possible score of the index was adjusted by subtracting the maximum score for the item from the maximum possible score of the index before calculating the percentage. If the sum of maximum scores for the omitted items exceeded 50 % of the maximum possible score for the index, or more than half of the items were omitted, the form was considered non-valid.

In the text and tables, data are presented as number and proportion or mean and standard deviation. Responsiveness was determined using anchor-based methods [30, 36, 37]. Sensitivity and specificity to discriminate between *improved* and *not or little changed* participants, based on the GRCS categorization, were determined for the ProFitMap-neck indices and NDI. To this end, receiver operating characteristic (ROC) curves were fitted for sample I and II separately to illustrate the discriminating ability of the indices [34]. From each ROC curve, the area under the curve (AUC) and its 95 % confidence interval was calculated and used as the primary measure of responsiveness. The NDI scale was inverted in this calculation to simplify the comparison. An area value of 0.5 indicates discrimination by chance, and a value of 1 indicates perfect discrimination [38]. For the second measure of responsiveness, we calculated the correlation (Spearman’s rho) between the GRCS anchor and change scores (index score after treatment—index score before treatment). Based on the ROC analyses, the minimal important change (MIC) was determined as the change score that showed the highest combination of sensitivity and specificity [39, 40]. All statistical analyses were performed in IBM SPSS Statistics 22.0 for Windows (IBM Corp, Armonk, NY).

## Results

The number of participants that completed the intervention was 89 in trial I and 104 in trial II. Four participants were excluded from the analysis because they rated <3 on GRCS (one participant from sample I and three from sample II). Of the remaining 88 participants in sample I, 47 rated an improvement in health after the intervention (i.e., 6 or 7 on the GRCS), and 41 were categorized as no or little change (i.e., rated 3, 4, or 5 on the GRCS). Of the remaining 101 participants in sample II, 54 rated an improvement and 47 did not do so. The characteristics and baseline measurements of the samples are shown in Table 1. The maximum possible score was reached at follow-up for five and six participants for the ProFitMap-neck function index and NDI, respectively. No participant reached the maximum possible score in any of the indices at baseline. Table 2 presents the change scores for each category in the two samples, including the proportion of missing items in the questionnaires.

**Table 1:** Characteristics and baseline measurements on all participants (*n* = 223)

	Sample I		Sample II	
	Total (*n* = 103)	Excluded (*n* = 15)	Total (*n* = 120)	Excluded (*n* = 19)
	Mean (SD) or Median (IQ-range)	Mean (SD) or Median (IQ-range)	Mean (SD) or Median (IQ-range)	Mean (SD) or Median (IQ-range)
Age (years)	52 (45–58)	46 (35–59)	53 (44–60)	54 (48–57)
Length (cm)	166 (6)	163 (4)	166 (6)	166 (5)
Weight (kg)	67 (61–79)	64 (57–78)	66 (60–74)	70 (63–74)
Pain duration (months)^M^	120 (60–216)	120 (42–192)	60 (24–123)	36 (10–120)
Pain intensity (NRS)^M^	5.0 (4.0–7.0)	7.0 (5.0–7.0)	5.0 (3.0–6.0)	5.0 (3.0–6.0)
Sick leave last 6 months (days)^M^	1.0 (1.0–1.0)	1.0 (1.0–2.0)	0.0 (0.0–0.0)	0.0 (0.0–1.0)
NDI^M^	72.0 (66.0–80.0)	68.0 (58.0–78.0)	78.0 (70.0–84.0)	76.0 (68.0–82.0)
ProFitMap-neck:				
Symptom-intensity index^T^	63.3 (11.5)	64.1 (11.0)	71.1 (9.1)	69.1 (12.0)
Symptom-frequency index^T^	57.2 (14.1)	56.5 (14.7)	65.9 (12.6)	60.4 (13.8)
Function index^T^	62.0 (13.5)	62.9 (12.6)	72.1 (11.8)	69.0 (13.7)
Total score^T^	60.9 (11.4)	61.6 (11.7)	70.3 (10.0)	66.9 (12.6)

Excluded incorporates those who discontinued the study and four respondents with PGIC < 3

The range for the scales in NDI and PFM is 0–100, NDI normalized

*SD* standard deviation, *IQ* inter-quartile range (25–75th percentile), *NRS* Numerical Rating Scale, *NDI* Neck Disability Index

^M^Mann–Whitney U-test of differences between total samples significant at 5 % significance level with Bonferroni correction

^T^ *T*-test of differences between total samples significant at 5 % significance level with Bonferroni correction

**Table 2:** Change scores for sample I and II, including the proportion of missing items in the questionnaires

	Sample I			Sample II		
	n	Mean change score (SD)	Missing items (%)	n	Mean change score (SD)	Missing items (%)
ProFitMap-neck						
Symptom-intensity index						
Improved	47	13.9 (11.1)	2	54	11.1 (8.1)	0
No or little change	41	5.0 (7.3)	0	47	1.8 (6.9)	0
Symptom-frequency index						
Improved	47	18.5 (11.7)	0	54	14.1 (9.8)	0
No or little change	41	6.2 (9.1)	0	47	3.2 (9.0)	0
Function index						
Improved	47	16.9 (13.0)	2	54	12.5 (10.4)	0
No or little change	41	7.0 (10.7)	3	47	3.5 (9.5)	0
Total score						
Improved	47	16.6 (11.3)	4	54	12.5 (8.0)	0
No or little change	41	6.2 (7.7)	3	47	3.0 (7.2)	0
NDI						
Improved	47	9.9 (8.2)	2	54	11.8 (7.4)	0
No or little change	41	2.8 (5.7)	0	47	1.5 (6.7)	0

*n* number of subjects, *SD* standard deviation, *NDI* Neck Disability Index

The AUC with 95 % confidence interval for the two samples is shown in Table 3. Overall, the ProFitMap-neck performed similarly to NDI, and the AUCs tended to be larger for sample II compared to sample I but the confidence intervals showed substantial overlap. Among the ProFitMap-neck indices, the function index had slightly lower AUC than the symptom indices.

**Table 3:** Area under the receiver operating characteristic curve (AUC) with 95% confidence interval for sample I and II

	Sample I		Sample II	
	AUC	95 % CI	AUC	95 % CI
ProFitMap-neck				
Symptom-intensity index	0.77	0.67–0.87	0.84	0.76–0.92
Symptom-frequency index	0.80	0.71–0.89	0.80	0.71–0.89
Function index	0.74	0.63–0.85	0.76	0.67–0.86
Total score	0.78	0.68–0.88	0.83	0.75–0.92
NDI	0.75	0.65–0.85	0.86	0.79–0.93

AUC reflects the ability of the scale to discriminate between improved and not or little changed participants. AUC = 0.5 indicates discrimination by chance, and a value of 1 indicates perfect discrimination [38]

*NDI* Neck Disability Index

In Table 4, the MIC and its corresponding sensitivity and specificity are shown for all indices in both samples. NDI had the lowest MIC in both samples. For sample I, this NDI-MIC value had the lowest sensitivity and specificity, but in sample II its sensitivity was higher. The highest combination of sensitivity and specificity was observed for the ProFitMap-neck symptom-intensity index in sample II. The highest MIC in both samples was obtained for the ProFitMap-neck symptom-frequency index. Overall, the MIC tended to be lower in sample II for all indices.

**Table 4:** Minimal important change (MIC) and its corresponding sensitivity and specificity for sample I and II

	Sample I			Sample II		
	MIC	Sensitivity	Specificity	MIC	Sensitivity	Specificity
ProFitMap-neck						
Symptom-intensity index	9.9	0.71	0.78	6.6	0.76	0.87
Symptom-frequency index	13.6	0.64	0.83	11.0	0.72	0.85
Function index	11.2	0.71	0.75	7.3	0.80	0.66
Total score	9.6	0.76	0.75	7.1	0.80	0.79
NDI	6.3	0.62	0.75	5.2	0.82	0.75

Sensitivity was defined as the rate of correctly classifying improved participants, and specificity as the rate of correctly classifying not or little changed participants. Minimal important change was determined as the change score that showed the highest combination of sensitivity and specificity

*NDI* Neck Disability Index

For sample I, Spearman’s rho between GRCS and the change scores of ProFitMap-neck and NDI ranged between 0.47 (ProFitMap-neck function index) and 0.59 (ProFitMap-neck symptom-frequency index). For sample II, the correlation ranged between 0.56 (ProFitMap-neck function index) and 0.72 (NDI). All correlations were significant (*p* < 0.05).

## Discussion

In the present study, we aimed to investigate the ProFitMap-neck performance by assessing its responsiveness, and compare that to NDI, in two samples of women with non-specific neck–shoulder pain. The results suggest that both measures possess similar ability to detect change in self-rated perceived health with AUC exceeding 0.7 which is a cut-off value that has been used to delineate adequate responsiveness [40–43]. While this was the first examination of responsiveness for ProFitMap-neck, several previous studies exist on this measurement property for NDI [30, 34, 36, 40–42, 44–47]. Most of these show results in concordance with the present study, except for two studies that found lower AUC for NDI (0.57 [36] and 0.59 [44]). In a review of measurement properties of eight neck-specific pain and disability questionnaires, where NDI but not ProFitMap-neck was included, it was concluded that NDI was one of two questionnaires that had better than limited evidence of responsiveness [5].

Correlation analyses between change scores and GRCSs showed significant associations for both ProFitMap-neck indices and NDI, which indicates that the GRCSs were valid anchors for our study [37, 48]. In contrast to the more general GRCS used in trial I, the GRCS in trial II explicitly expressed neck–shoulder problems and may therefore have better construct validity as an external anchor [32, 49]. This could have affected our results; however, correlations were only slightly higher in trial II, and earlier findings of similar reliability for questions on general perceived recovery compared to perceived change in neck pain [50] indicate that both types of questions could be used. Global rating of change scales of general perceived recovery seem to be the most common external anchors (see e.g. [30, 36, 40, 41, 46, 47]).

Minimal important change of normalized values in the two samples examined ranged between 6.6 and 13.6 % for the ProFitMap-neck indices and was 6.3 and 5.2 % for the NDI. The symptom-frequency index had the highest MIC in both samples. This may reflect the often existing temporal variation of symptoms in neck pain individuals [7, 51]. The symptom-frequency index had also the highest measurement error in the previous validation study of ProFitMap-neck [12]. However, pain frequency may still be important to measure in chronic pain clinical trials since temporal aspects of pain have shown to be a valid dimension discerned from pain intensity, therefore recommended as an outcome [31]. The MICs obtained for NDI are rather low compared with previous studies in chronic neck pain, showing a range of 5–19 % [30, 34, 36, 40–42, 44, 47, 52].One explanation for this may be the low mean NDI baseline scores of 28 and 23 NDI% in sample I and II, respectively. Association between NDI baseline scores and MIC was recently demonstrated, showing larger MIC for those above (i.e., with higher disability) compared to those below (i.e., with lower disability) median baseline score [42, 44, 52]. The same effect of baseline values on MIC in neck pain patients was also shown for pain intensity numerical rating scale [53], but not for Neck Pain Disability Scale [42]. In the comparison of MIC values of NDI and the ProFitMap-neck indices, the latter were slightly higher. However, the combination of sensitivity and specificity for the MICs was higher in all ProFitMap-neck indices in sample I and in the majority of the ProFitMap-neck indices in sample II. The comparison of the MIC of ProFitMap-neck with MIC of other neck-specific questionnaires beside NDI is hampered by the low number of studies and differing methodology to determine MIC. For comparable studies, Neck Pain and Disability Scale [41, 42] and Neck Bournemouth Questionnaire [54] had MIC of similar magnitude as ProFitMap-neck, whereas MIC reported for the Core Outcome Measure Index summary score was higher (20 and 27 %) [55, 56].

Methods to determine MIC can be sorted into anchor-based or distribution-based approaches. Distribution-based methods are conceptually different in being based on statistical characteristics of the sample distribution. These methods rather deal with minimal detectable change than any indication of the *importance for the patient* of the observed change, which is the ground for anchor-based methods [48, 57, 58]. In the current study, we used anchor-based methods for determining responsiveness and MIC, thereby considering patient perception as a key factor for the MIC [59] in accordance with its conceptual definition [17].

However, the reliance of anchor-based methods poses several challenges. The first concerns the validity of the external anchor. In line with many other studies [30, 34, 36, 40–42, 44–47, 53, 60], we used GRCS as the external anchor to discern improved versus no or little change. This method has been criticized, one reason being recall bias [32]. The COSMIN (Consensus-based Standards for the selection of health status Measurement Instruments) checklist points out that GRCS should not be regarded as a gold standard, and suggests that no gold standard exists for patient-reported outcomes except for longer versions of the same outcome as the one under test [17]. However, the same checklist recommends using a GRCS of the same construct as the instrument under study as a useful comparator with high face validity, and evidence supports the use of GRCS with 7–11 response alternatives [32]. Also, in a review on methodological quality of neck questionnaire studies, GRCS was deemed appropriate and the best criterion available [6]. A second challenge of anchor-based methods, brought up by de Vet and co-workers [57], is that they do not include any aspect of measurement precision, thereby leaving out information whether the MIC lies within measurement error, i.e., is smaller than minimal detectable change, of the tested scale or not. The MIC of the ProFitMap-neck indices established in the present study was smaller than the smallest detectable change earlier determined from test–retest of 45 subjects with non-specific neck pain [12]. The same situation applies to our result on the MIC for NDI, i.e., they were smaller than minimal detectable change observed in most other studies. As a matter of fact, MIC was always smaller than minimal detectable change in NDI (see compilation, Table 1 in [52]), meaning that MIC may be confounded with measurement error [58]. Thus, using minimal detectable change instead of MIC as cut-off in NDI and ProFitMap-neck increases the certainty of that measurement error will be exceeded and should therefore be the choice when a high rate false positive (low specificity) should be avoided. The MIC, expressed as the optimal point on the ROC curve for high sensitivity and specificity equally weighted, may be used as an alternative cut-off in situations where a low rate of false negative (high sensitivity) is equally important. Finally, the use of anchor-based methods to determine responsiveness is not suitable if the proportion of improved versus not improved are severely skewed with only few individuals in one category [61]. This was, however, not the case in either sample (Table 2).

Limitations of the study include the long time period of 12 weeks between measurements which may increase recall bias for the GRCS questions. Another aspect to consider is the generalizability of the results to other women with subacute and chronic non-specific neck pain. The recruitment procedure in both trials was partly done by advertising [22, 23], and samples should therefore be considered as convenience samples which constituted of women with relatively mild pain and disability. This may reduce the generalizability of results. Also, findings cannot be generalized to men with neck pain. A further limitation is that the interventions given could potentially have influenced the MIC differently, but separate analyses of each intervention group were not possible due to small group sample sizes. Finally, the small differences between trial I and II in respect of the inclusion criteria and wordings of the external anchors, and the differences in characteristics and baseline measurements, made us unwilling to pool the data into one sample. This could be seen as a drawback due to reduced sample size, but the number of participants in each sample was most likely adequate for our purpose [62]. With that in mind, the separate samples used could be regarded a strength of the study since confirmation of responsiveness across samples is recommended [37].

## Conclusions

This study extends the knowledge of measurement properties of the ProFitMap-neck questionnaire by endorsing its validity for change scores in two groups of women with non-specific neck–shoulder pain. In both groups, adequate ability to discriminate between improved and not or little changed participants was demonstrated and values of important change presented. The responsiveness of the ProFitMap-neck was similar to that of NDI which, in turn, was similar to earlier findings corroborating NDI and ProFitMap-neck as responsive measures. Continuing future validation of the ProFitMap-neck is warranted and should include other neck pain conditions as well as men.
